# *CBNplot*: Bayesian network plots for enrichment analysis

**DOI:** 10.1093/bioinformatics/btac175

**Published:** 2022-03-25

**Authors:** Noriaki Sato, Yoshinori Tamada, Guangchuang Yu, Yasushi Okuno

**Affiliations:** Department of Biomedical Data Intelligence, Graduate School of Medicine, Kyoto University, Kyoto 606-8507, Japan; Department of Biomedical Data Intelligence, Graduate School of Medicine, Kyoto University, Kyoto 606-8507, Japan; Innovation Center for Health Promotion, Hirosaki University, Aomori 036-8562, Japan; Department of Bioinformatics, School of Basic Medical Sciences, Southern Medical University, Guangzhou 510515, China; Department of Biomedical Data Intelligence, Graduate School of Medicine, Kyoto University, Kyoto 606-8507, Japan; RIKEN Advanced Institute for Computational Sciences, Hyogo 650-0047, Japan

## Abstract

**Summary:**

When investigating gene expression profiles, determining important directed edges between genes can provide valuable insights in addition to identifying differentially expressed genes. In the subsequent functional enrichment analysis (EA), understanding how enriched pathways or genes in the pathway interact with one another can help infer the gene regulatory network (GRN), important for studying the underlying molecular mechanisms. However, packages for easy inference of the GRN based on EA are scarce. Here, we developed an R package, *CBNplot*, which infers the Bayesian network (BN) from gene expression data, explicitly utilizing EA results obtained from curated biological pathway databases. The core features include convenient wrapping for structure learning, visualization of the BN from EA results, comparison with reference networks, and reflection of gene-related information on the plot. As an example, we demonstrate the analysis of bladder cancer-related datasets using *CBNplot*, including probabilistic reasoning, which is a unique aspect of BN analysis. We display the transformability of results obtained from one dataset to another, the validity of the analysis as assessed using established knowledge and literature, and the possibility of facilitating knowledge discovery from gene expression datasets.

**Availability and implementation:**

The library, documentation and web server are available at https://github.com/noriakis/CBNplot.

**Supplementary information:**

Supplementary data are available at *Bioinformatics* online.

## 1 Introduction

Identification of differentially expressed genes (DEGs) between conditions, especially disease conditions, and subsequent enrichment analysis (EA) can help infer the biological basis of the differences. In EA, understanding how pathways or genes in a pathway interact with one another can help infer the gene regulatory network (GRN), which is important for studying the underlying molecular mechanisms. However, packages for easily inferring the GRN based on EA are scarce.

Here, we developed *CBNplot*, an R package that explicitly uses curated biological pathway information with EA to construct the Bayesian network (BN). The unique aspects of the package are probabilistic reasoning and visualization using EA results from *clusterProfiler* (Wu *et al.*, 2021) family and core functions from *bnlearn* (Scutari, 2010).

## 2 Implementation

The core features are convenient wrapping for structure learning, visualization of the BN from EA results and comparison with reference networks using *graphite* (Sales *et al.*, 2012). For structure learning, the inference is performed using the bootstrap-based approach based on R library *bnlearn*. Various score-based and constraint-based algorithms can be used to quantitatively calculate the degree of relatedness between genes or pathways with parallel computing according to the functions of *bnlearn* (Imoto *et al.*, 2002; Scutari, 2010). For genes, the preprocessed expression profile was used to infer the BN, and for pathways, the eigengene in the pathway was used as the pathway expression value (Oldham *et al.*, 2006; Foroushani *et al.*, 2017). The hub genes and edges with high confidence for direction can be further visualized in the network.

Users can additionally connect the dependency score provided by the Dependency Map (DepMap) to the inferred network, especially for cancer-related research (Tsherniak *et al.*, 2017). DepMap calculates the dependency score by genome-scale loss-of-function screens in cancer-related cell lines using the CRISPR/Cas9 system and RNA interference. The R package *depmap* is used to obtain DepMap data (Killian and Gatto, 2021). Users can specify their cell line or lineage of interest.

## 3 Results

**Fig. 1.**
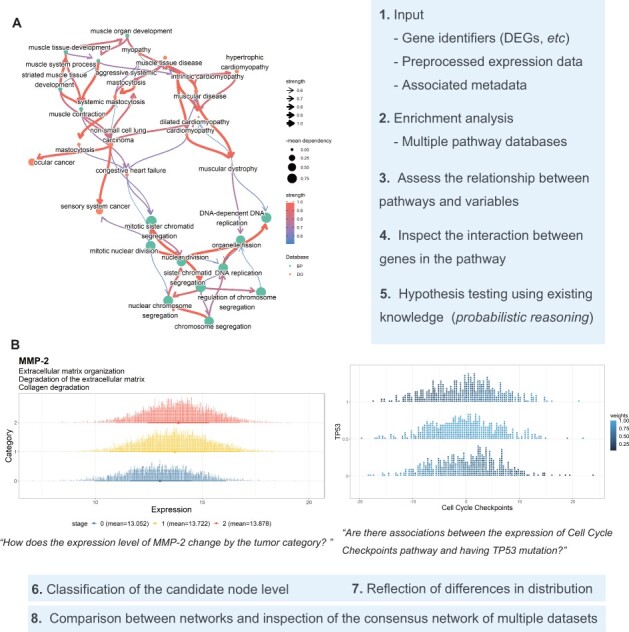
The *CBNplot* workflow. Representative plots made using *CBNplot* are shown. (**A**) The Bayesian network (BN) plot showing the relationship between pathways significantly related to bladder cancer from the gene and disease ontology. (**B**) The dotplot showing conditional distribution of the expression of *MMP-2* by logic sampling, conditional on the evidence of tumor category. The BN was based on genes belonging to the three pathways in the subtitle.

We illustrate the application using the RNA-Seq data of bladder cancer deposited in the Gene Expression Omnibus database under the accession identifier GSE133624 (Chen *et al.*, 2019) and data from The Cancer Genome Atlas Urothelial Bladder Carcinoma (TCGA-BLCA) and its associated clinical variables (Cancer Genome Atlas Research Network *et al.*, 2013; Colaprico *et al.*, 2016; Robertson *et al.*, 2017). We used DESeq2 to identify DEGs in GSE133624, which were passed to *clusterProfiler* or *ReactomePA* to infer the differentially expressed biological pathways (Jassal *et al.*, 2020; Love *et al.*, 2014; Yu and He, 2016). The figure was derived from *bnpathplot*, a core function of the library performing network inference by the biological pathways as nodes and using the pathway expression values (Fig. 1A).

The inferred networks using EA results can be verified through probabilistic reasoning and classification. We used TCGA-BLCA data and the over-representation analysis results of *ReactomePA* inferred from the DEGs of GSE133624. We obtained significantly enriched pathways related to the matrix metalloproteinase-2 (*MMP-2*) gene and constructed the network using the genes in the top three representative pathways as nodes and their expression values by the function *bngeneplot*. Age and factorized tumor category were included as clinical variables. We sampled the conditional distribution of *MMP-2* expression by setting the tumor category as evidence using the wrapper function of *cpdist* in *bnlearn*. The resulting distribution of *MMP-2* for each category (Fig. 1B) was plotted using the library *ggdist* (Kay, 2020). The predicted expression considering the network in *MMP-2*-related pathways increased with tumor category (Kanayama *et al.*, 1998). The application result of the classification of a clinical variable using BN inferred from gene expression values is depicted in Supplementary Text S1 and Supplementary Figures S1 and S2. The runtime and the assessment of network stability are summarized in Supplementary Table S1 and Supplementary Figure S3.

## 4 Conclusion

*CBNplot* combines EA results from curated biological pathways, BN inference, probabilistic reasoning and classification using *bnlearn*, with subsequent visualization. The package aims to infer the BN from the EA results in the defined sets of biological pathways that include basically less than a hundred genes, thus alleviating the number of nodes for the inference in the general computer resources. As the inference of BN from hundreds of nodes is less reliable when the sample size is low, the result should be interpreted carefully. It can highlight interactions between genes and pathways and benefit the identification of candidate genes of interest or hypothesis testing through knowledge mining and visualization.

## Supplementary Material

btac175_Supplementary_Data
