# Supplementary material for: CBNplot: Bayesian network plots for enrichment analysis
Source: Bioinformatics. 2022 Mar 25;38(10):2959–60. doi: 10.1093/bioinformatics/btac175 (PMC9113354; doi:10.1093/bioinformatics/btac175)
Supplement: btac175_Supplementary_Data [file btac175_supplementary_data.docx]

**Supplementary Table 1. Runtime in milliseconds**

| **Type of run** | **Min** | **Mean** | **Max** |
| --- | --- | --- | --- |
| Correlation (gene number = 96) | 111.8 | 114.6 | 117.0 |
| Correlation (gene number = 44) | 107.3 | 109.4 | 111.0 |
| *bngeneplot* (gene number = 96) | 64670.9 | 68340.0 | 70968.5 |
| *bngeneplot* (gene number = 44) | 5059.8 | 5074.8 | 5084.8 |

The sample size was 433. The performance was assessed using the R library *microbenchmark* by performing the function thrice on four threads. The computational processing unit used was an AMD Ryzen 9 3950X 16-Core Processor on the Windows 10 operating system and R version 4.1.0.

**Supplementary Text 1. The classification of clinical variables**

Bayesian networks (BNs) can be used to classify clinical variables. We inferred BNs of genes in the reactomes at the significance threshold of the corrected p-value of 1e-5 using a *bngeneplot* function in *CBNplot*, including age, gender, tumor category, and presence or absence of TP53 mutation as variables (Knijnenburg et al., 2018). Fivefold cross-validation was performed using the *caret* package (Kuhn, 2008). BNs were estimated and fitted using a training dataset. The performance was assessed by the testing dataset using the area under receiver operating characteristic curves (AUROC), calculated and plotted using *pROC* (Robin et al., 2011). The inferred network that did not meet the directed acyclic assumption and had TP53 nodes without parents were excluded. As a result, the network of “DNA strand elongation” achieved the highest average AUROC of 0.769 ± 0.033. We visualized the ROC plot of the three pathways with the highest average AUROC (Supplementary Figure 1). Importantly, using the network including the clinical variables, we can reflect the differences in the distribution means to the network, calculated by setting the probability of clinical variables at 0 and 1 (Supplementary Figure 2).

**References**

Knijnenburg,T.A. *et al.* (2018) Genomic and molecular landscape of DNA damage repair deficiency across The Cancer Genome Atlas. *Cell Rep.*, **23**, 239-254.e6.

Kuhn,M. (2008) Building predictive models in R using the caret package. *J. Stat. Software, Articles*, **28**, 1–26.

Robin,X. *et al.* (2011) pROC: an open-source package for R and S+ to analyze and compare ROC curves. *BMC Bioinformatics*, **12**, 77.

**Supplementary Figure 1. The classification performance using the Bayesian network inferred using *CBNplot*
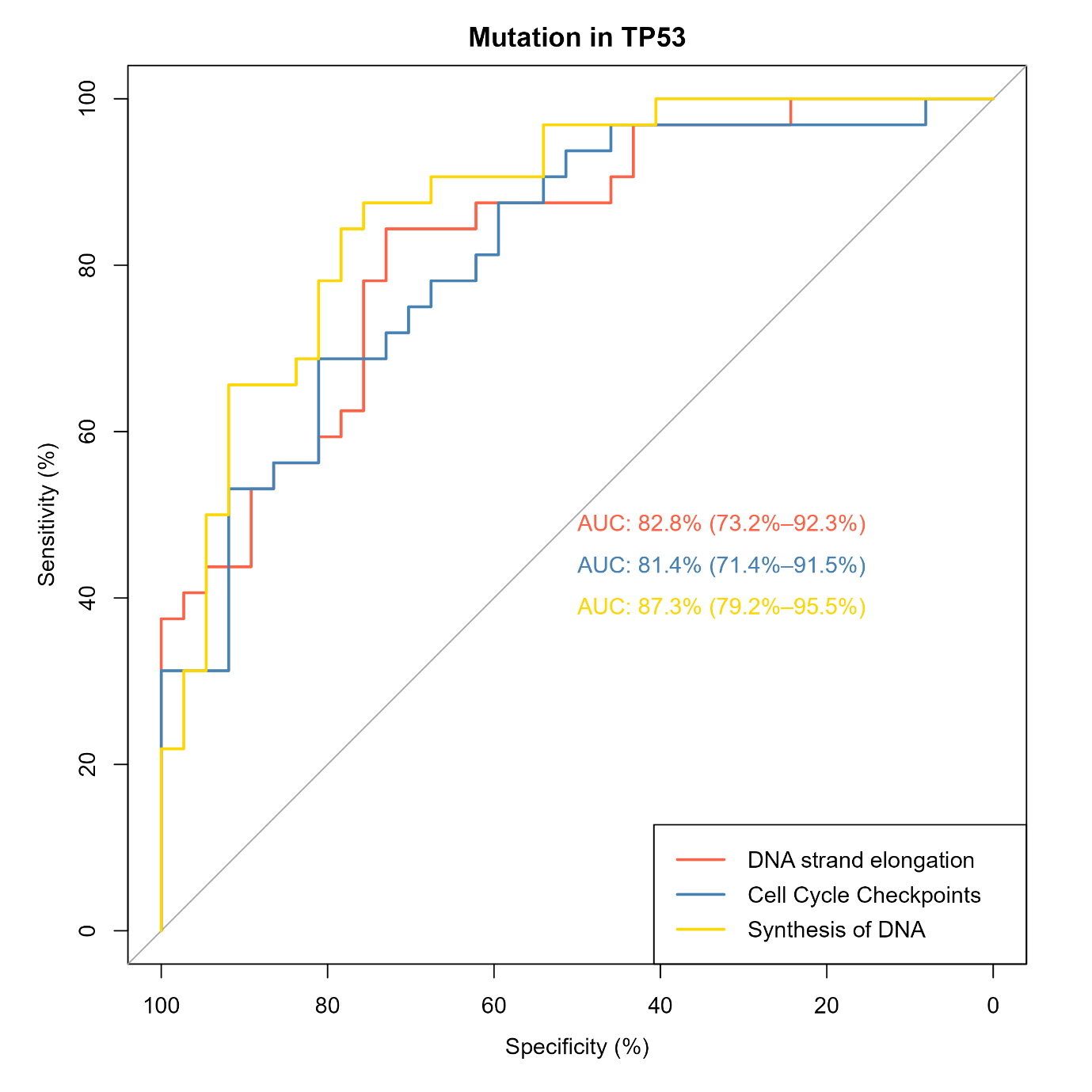
**

The receiver operating characteristic curves (ROC) plot depicting classification performance of the TP53 gene mutation using the expression values of biological pathways is shown. The values of the area under the ROC are shown with confidence intervals.

**Supplementary Figure 2. The gene network of DNA replication pathway representing the differences by clinical variables using probabilistic reasoning
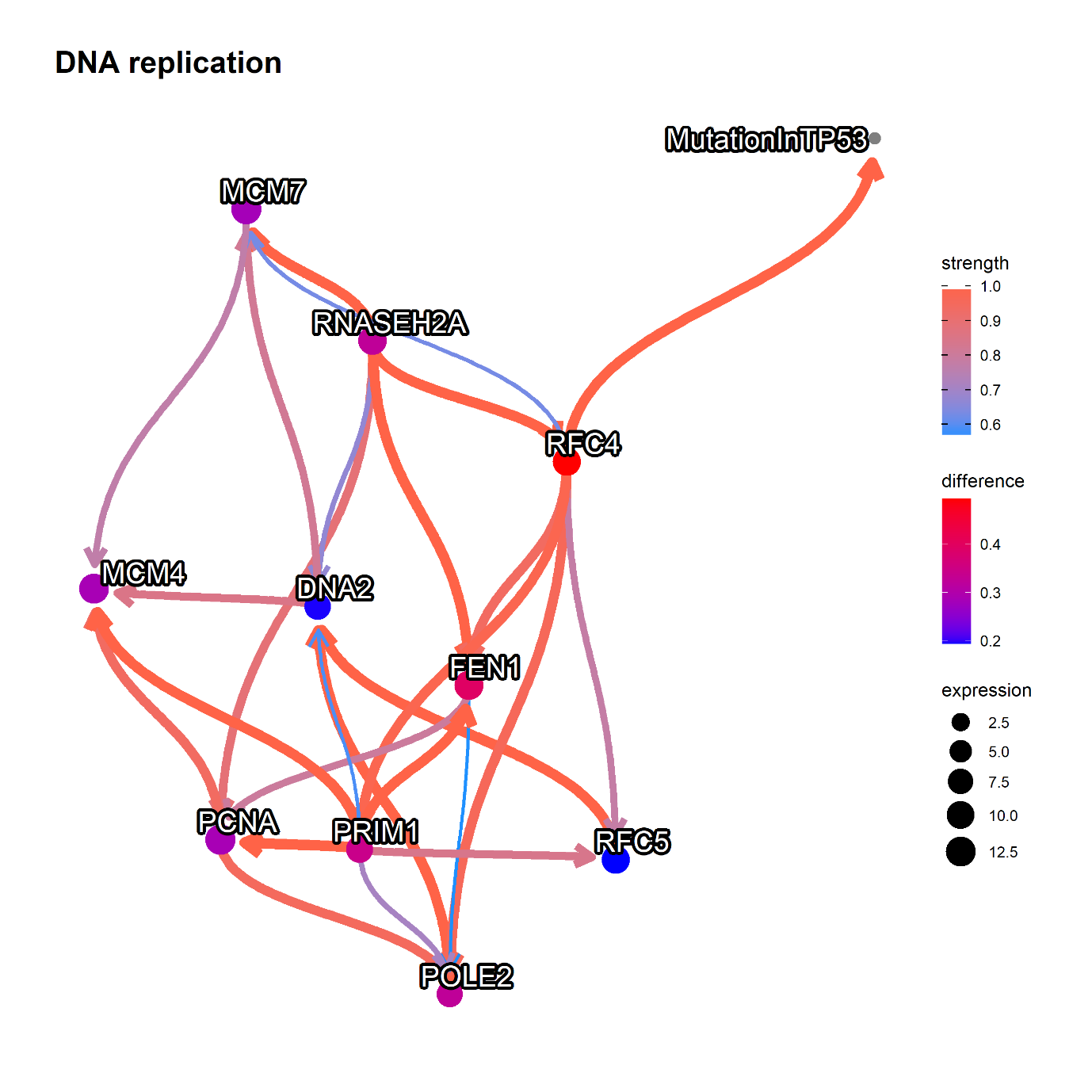
**The network was inferred using the differentially expressed genes derived from GSE133624 and gene expression data of The Cancer Genome Atlas Urothelial Bladder Carcinoma. The interaction between genes inside the DNA replication pathway is shown. The node color represents the differences in the mean of the distribution when the value of the variable of whether the sample is harboring the mutation in TP53 (MutationInTP53) is set to 0 and 1.

**Supplementary Figure 3. The assessment of the inferred network stability**

**
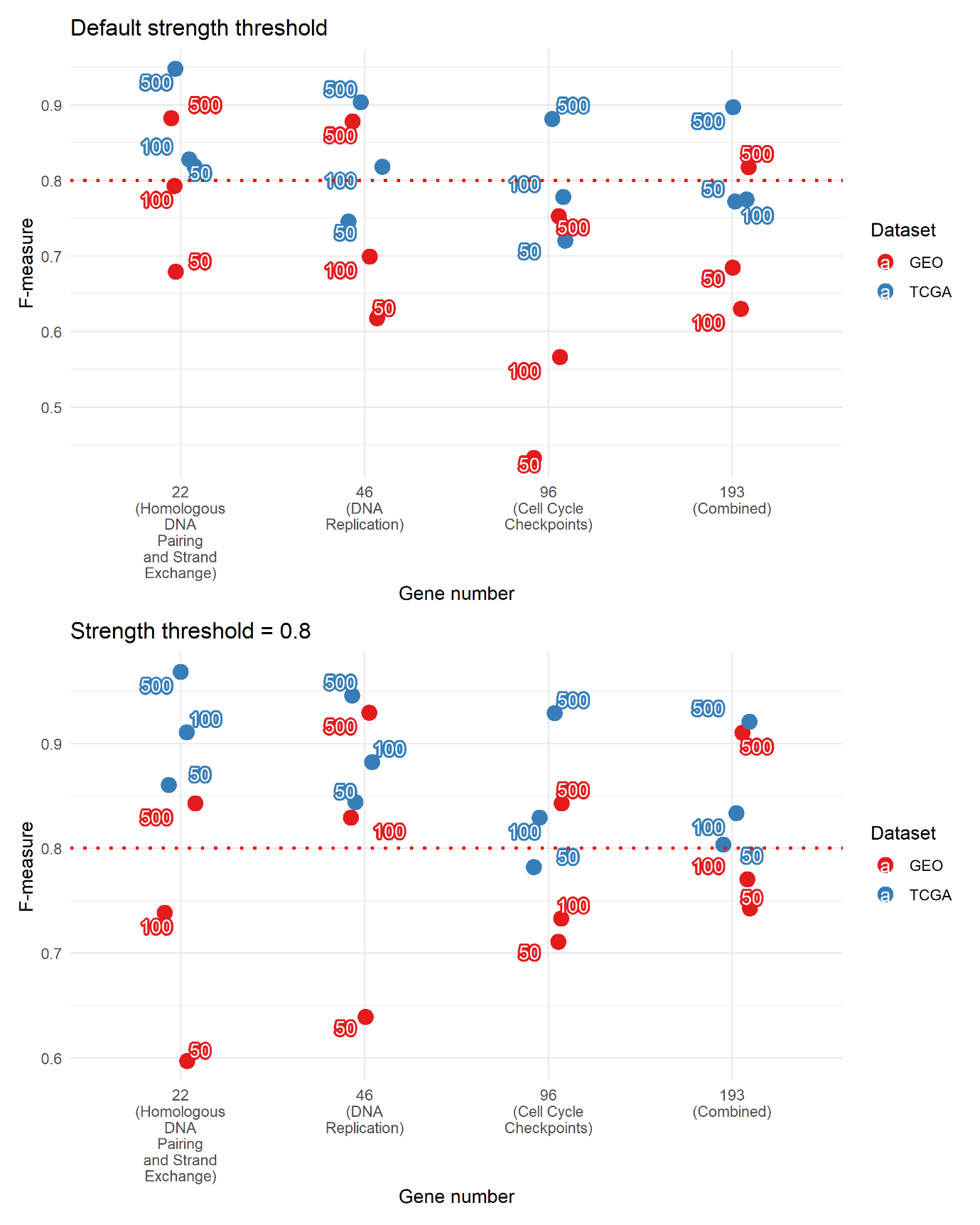
**

Bayesian network was inferred five times using the Hill-Climbing algorithm from the dataset consisting of corresponding gene numbers, and the mean of F-measures for each pair of the network is plotted. The point label represents the bootstrap number. The first row corresponds to the default strength threshold and the second row represents the strength threshold of 0.8. The dotted line indicates the value of 0.8. GEO, GSE133624; TCGA, The Cancer Genome Atlas Urothelial Bladder Carcinoma; Combined, genes in the top-10 enriched pathway combined.
